# Wnt/β-Catenin Signaling Regulates CXCR4 Expression and [^68^Ga] Pentixafor Internalization in Neuroendocrine Tumor Cells

**DOI:** 10.3390/diagnostics11020367

**Published:** 2021-02-22

**Authors:** Alexander Weich, Dorothee Rogoll, Sophia Gawlas, Lars Mayer, Wolfgang Weich, Judit Pongracz, Theodor Kudlich, Alexander Meining, Michael Scheurlen

**Affiliations:** 1 Department of Internal Medicine II, Gastroenterology, University Hospital Würzburg, 97080 Würzburg, Germany; Rogoll_D@ukw.de (D.R.); gawlas_s@ukw.de (S.G.); wolfgang.weich@gmx.de (W.W.); kudlich_t@ukw.de (T.K.); meining_a@ukw.de (A.M.); Scheurlen_m@ukw.de (M.S.); 2 Department of Nuclear Medicine, University Hospital Würzburg, 97080 Würzburg, Germany; mayer_l3@ukw.de; 3 Department of Pharmaceutical Biotechnology, Faculty of Pharmacy, University of Pecs, 7630 Pecs, Hungary; pongracz.e.judit@pte.hu

**Keywords:** neuroendocrine tumor, NET, Wnt, β-catenin, CXCR4, [^68^Ga] Pentixafor, BON-1, QGP-1, MS-18

## Abstract

Loss of Somatostatin Receptor 2 (SSTR2) expression and rising CXC Chemokine Receptor Type 4 (CXCR4) expression are associated with dedifferentiation in neuroendocrine tumors (NET). In NET, CXCR4 expression is associated with enhanced metastatic and invasive potential and worse prognosis but might be a theragnostic target. Likewise, activation of Wnt/β-catenin signaling may promote a more aggressive phenotype in NET. We hypothesized an interaction of the Wnt/β-catenin pathway with CXCR4 expression and function in NET. The NET cell lines BON-1, QGP-1, and MS-18 were exposed to Wnt inhibitors (5-aza-CdR, quercetin, and niclosamide) or the Wnt activator LiCl. The expressions of Wnt pathway genes and of CXCR4 were studied by qRT-PCR, Western blot, and immunohistochemistry. The effects of Wnt modulators on uptake of the CXCR4 ligand [^68^Ga] Pentixafor were measured. The Wnt activator LiCl induced upregulation of CXCR4 and Wnt target gene expression. Treatment with the Wnt inhibitors had opposite effects. LiCl significantly increased [^68^Ga] Pentixafor uptake, while treatment with Wnt inhibitors decreased radiopeptide uptake. Wnt pathway modulation influences CXCR4 expression and function in NET cell lines. Wnt modulation might be a tool to enhance the efficacy of CXCR4-directed therapies in NET or to inhibit CXCR4-dependent proliferative signaling. The underlying mechanisms for the interaction of the Wnt pathway with CXCR4 expression and function have yet to be clarified.

## 1. Introduction

Neuroendocrine neoplasia (NEN) is a heterogeneous group of tumors with increasing incidence originating from cells of the neuroendocrine system. Two thirds of all neuroendocrine tumors develop in the gastrointestinal tract or the pancreas [1,2].

Neuroendocrine carcinomas (NEC) are undifferentiated tumors with high proliferation rates (Ki-67 > 80%), whereas the histologically differentiated neuroendocrine tumors (NET) typically show proliferation rates between 1% and 50%. NET retain histological characteristics and functional properties of the original neuroendocrine tissue. This may result in tissue-specific expression of somatostatin receptors [1]. The clinically most relevant receptor in NET is somatostatin receptor type 2 (SSTR2), which is expressed by most well-differentiated NET and represents the pivotal diagnostic and therapeutic target in G1/G2 NET [3].

While the expression of SSTR, and especially of SSTR2, is associated with a higher grade of differentiation in NET, the expression of the C-X-C motif chemokine receptor 4 (CXCR4) is a feature of dedifferentiation in these tumors. CXCR4 and its ligand CXCL12 (also known as SDF-1α) play key roles in hematopoiesis and lymphoid tissue architecture and in cardiogenesis, vascular formation, and neurogenesis [4]. Pathologic expression of CXCR4 has been described in various solid tumors and hematologic malignancies. It is linked to an enhanced metastatic and invasive potential and a worse prognosis [4,5]. Because of its high expression rate, CXCR4 is considered a potential target for endoradiotherapy (ERT), targeted molecular therapy, and immunotherapy in some malignant tumors [6]. Several CXCR4 antagonists have been developed, e.g., AMD3100 (plerixafor), AMD3465, or TN14003. Their antiproliferative effects were demonstrated both in vitro and in animal experiments [7,8], and some have entered clinical trials [9]. For noninvasive assessment of CXCR4 expression and subsequent chemokine-directed endoradiotherapy, the theranostic agents [^68^Ga]Pentixafor and [^177^Lu]/[^90^Y]Pentixather were developed [10,11,12]. In tumor samples of NET Patients, a rise in CXCR4 expression and a concomitant decrease in SSTR2 expression were found with increasing degrees of malignancy [3]. This observation was confirmed noninvasively by our group in vivo in a retrospective triple tracer study with [^68^Ga]Pentixafor PET CT, [^68^Ga]DOTATOC PET CT, and [^18^F]FDG PET CT [13].

Deregulation of Wnt/β-catenin signaling has been demonstrated in NET. In approximately 25% of NET samples, an accumulation of β-catenin in the cytoplasm and/or nucleus as well as APC and β-catenin mutations were found to lead to constitutive activation of this pathway [14]. Both CXCR4/CXCL12 signaling as well as Wnt signaling have been shown to induce cell migration, expression of Epithelial–Mesenchymal Transition (EMT) markers, and proliferation in neuroendocrine tumors in vitro [14,15,16].

An interaction between the Wnt pathway and CXCR4-mediated signaling has been documented in malignant diseases, where the canonical Wnt pathway may be activated via CXCR4 signal transduction [5,17]. On the other hand, an inverse mechanism is present in dentate gyrus progenitor cells, where Wnt activation induces CXCR4 gene expression [18].

So far, data on the influence of canonical Wnt signaling on the expression of CXCR4 in NEN are missing completely. We therefore investigated if CXCR4 expression as well as receptor-associated functions can be modulated by either Wnt inhibition or stimulation in NET cells.

## 2. Materials and Methods

### 2.1. Cell Culture

The human cell lines BON-1 and QGP-1, originating from a pancreatic NET, were a gift from Prof. C. Grötzinger (Department of Hepatology and Gastroenterology, Charité-Universitätsmedizin, Berlin, Germany). We tested both cell lines as mycoplasm-free. Using PCR-single-locus-technology with PowerPlex 21 Kit from Promega (done by eurofins, Ebersberg, Germany), the identities of the two cells lines were authenticated. The identity of QGP-1 was confirmed by the Leibniz-Insitute (DSMZ, Braunschweig, Germany). There were no reference profiles for BON-1 present in DSMZ, but the Short Tandem Repeat (STR) profile was uniform and without evidence of contamination with another cell population. The results of our STR profiling correspond to the data published elsewhere [19]. The cell line MS-18 is a primary cell culture from a metastasized NEC of the rectum. Written consent for Biobank storage and the scientific use of biopsy material was obtained from the patient. The cell line was tested as mycoplasm-free. Detailed molecular characterization of the cell line is not yet available but will be published soon.

The QGP-1 cells were cultured in Dulbecco’s Modified Eagle’s Medium (DMEM) (Sigma Aldrich, St. Louis, MO, USA) + RPMI-1640-Medium (Gibco, Carlsbad, CA, USA) supplemented with fetal bovine serum (FBS; 10% *v*/*v*) (FBS Superior, Biochrom, Berlin, Germany) and Pen/Strep (1 × 10^5^ u/L) (Gibco). The BON-1 cells were cultured in DMEM + Nutrient Mixture F12 Ham (Sigma Aldrich) supplemented with FBS (10% *v*/*v*) and Pen/Strep (1 × 10^5^ u/L). These cells were cultured in 75 cm^2^ culture flasks (Greiner bio-one B.V, Kremsmünster, Austria) at 37 °C in a 5% CO_2_ humidified incubator and passaged with trypsin (Trypsin EDTA 0.05%, Gibco) when confluent. MS-18 cells were cultured in T75 primaria Tissue culture flasks (Corning, NY, USA) in Dulbecco’s modified eagles medium/F12 medium (Gibco) supplemented with Nu-Serum (Corning) and Insulin-Transferrin-Selenium (Gibco) at 37 °C in a 5% CO_2_ humidified incubator and passaged with Accumax solution (Sigma Aldrich) when confluent. The media of all three cell lines were supplemented every 3–4 days until a maximum of 15 passages.

5-aza-cytidine (5-aza-CdR) was obtained from Sigma Aldrich, quercetin was obtained from Acros Organics (Fair Lawn, NJ, USA), and niclosamide and LiCl were obtained from Sigma Aldrich.

EC50 for the inhibition of cell growth were determined for all Wnt modulators using the CCK8 kit (Sigma Aldrich). The cells were cultured in 96-well plates (7500 cells per well). Under these conditions, EC50 was 0.5 µM for 5-aza-CdR (incubation time 168 h), 75 µM for quercetin (incubation time 72 h), 10 µM for niclosamide (incubation time 6 h), and 7.5 mM for LiCl (incubation time 6 h) in BON-1 and QGP-1 cells. In MS-18 cells, EC50 for 5-aza-CdR and niclosamide corresponded to those of BON-1 and QGP-1 at identical incubation times. EC50 with quercetin was not reached in MS-18 cells with 75 µM and was 7.5 mM for LiCl (incubation time 24 h). The incubation times were chosen according to the optimal effect on CXCR4 mRNA expression as determined in a series of preliminary experiments for each drug.

Since 5-aza-CdR is not stable in media, we exchanged the incubation medium every 3 days. As described elsewhere [20], we used a dose of 0.1 µM and an incubation time of 168 h, which is well below the EC50 or therapeutic plasma concentrations. The conditions for the other drugs in our experiments were quercetin 50 µM, incubation time 24 h; niclosamide 7.5 µM, incubation time 6 h; and LiCl 5 mM, incubation time 4 h. The drug concentrations for all Wnt inhibitors used in our studies were well below the respective EC50 concentrations as well as the plasma concentrations that are achieved in the clinical setting [21,22,23]. Since the Wnt activator LiCl stimulates proliferation in BON-1, QGP-1, and MS-18 cells, the EC50 concentration for LiCl was much higher than the plasma concentrations in the clinical setting [24]. Additionally, in a study on mesenchymal stem cells, concentrations of 10–20 mM were required to affect CXCR4 expression [25]. The chosen concentration of 5 mM is above therapeutically used plasma levels but below the EC50 concentration.

### 2.2. RNA Isolation and Quantitative RT-PCR 

Quantitative RT-PCR (qRT-PCR) was performed as previously described [26]. In brief, total RNA was isolated using NucleoSpin RNA (Machery-Nagel, Düren, Germany) and cDNA was synthesized from 1 µg of total RNA with iScript (BioRad, Hercules, CA, USA) according to the manufacturer´s instructions. qRT-PCR was performed using the Absolute QPCR Mix (Thermo scientific, Waltham, MA, USA), and detection was conducted using an AB ViiA^TM^7 Real-time PCR System (Life Technologies, Carlsbad, CA, USA). TaqMan gene expression assays for qRT-PCR and endogenous controls were purchased from Applied Biosystems, Foster City, USA (for details, see Supplementary Table S1).

### 2.3. Immunohistochemistry

To assess the expression status of CXCR4 in BON-1, QGP-1, and MS-18 cells, we prepared cytospins with a total of 5 × 10^5^ cells per spin. The antibodies for CXCR4 (dilution 1:200) were applied using the super-sensitive streptavidin–peroxidase antiperoxidase technique (Biogenex, Fremont, CA, USA). For details on the antibodies, see Supplementary Table S1. The slides were evaluated, and additionally, the percentage of CXCR4 positive cells was calculated before and after Wnt modulator exposure.

### 2.4. Western Blot

Western blot analysis was performed as described previously [27]. In brief, the cells were seeded at 5 × 10^5^ cells per well (6 well plates, Greiner-Bio-one) and incubated with the individual drugs as described above at 37 °C and 5% CO_2_. Cells were washed twice with Dulbecco’s Phosphate Buffered Solution (DPBS, Gibco) and harvested in ice-cold lysis buffer (Ripa buffer, Thermo Scientific, supplemented with Protease Inhibitor Cocktail, Thermo Scientific). The lysates were homogenized, and the protein content was determined enzymatically (BCA Protein Assay, Thermo Scientific). An SDS sample buffer (4× Laemmli Sample Buffer, BioRad, supplemented with Bond Breaker, Thermo scientific) was added, and heat denaturation was performed at 95 °C for 3 min, except for the probes for CXCR4, which must not be boiled. Twenty micrograms of the samples were loaded on SDS-polyacrylamide gels (10% polyacrylamide) and size fractionated by electrophoresis.

For immunoblotting, the proteins were transferred onto immobilon-P PVDF transfer membranes (Merck, Millipore, Burington, MA, USA). The membrane was blocked with 5% nonfat dried milk (BioRad) in Tris Buffer Solution (TBS) (140 mM NaCl, 10 mM Tris-HCl, pH 7.4) and incubated with the primary antibodies (for details, see Supplementary Table S1) for 2 h at room temperature or overnight at 4 °C. After intense washes with TBS, secondary antibodies (for details, see Supplementary Table S1) were added and incubated for 1 h. Bound antibodies were detected with a chemiluminescence detection system (ECL Clarity Western ECL Substrate, BioRad).

### 2.5. CXCR4 Receptor Uptake

BON-1, QGP-1, or MS-18 cells were seeded in 24 well plates (Greiner bio-one) (2 × 10^5^ per well) and incubated with Wnt modulators under the conditions described above. Subsequently, the cells were washed twice with washing medium (DMEM + 30 mM HEPES, 0.2% Bovine Serum Albumin (BSA), 2 mM L-glutamine, 1 mM Na-pyruvate, and 2% Pen/Strep). Four wells per treatment (duplicate incubation with 0.37 MBq [^68^Ga] Pentixafor) were incubated for 1 h/2 h at 37 °C. After incubation, the cells were washed twice with ice-cold washing medium. The cells were extracted with 0.1 M NaOH to obtain the internalized fraction. By Wizard2 2480 (Perkin Elmar, Waltham, MA, USA), internalized counts per minute were determined in each treatment condition. The values were corrected for differences in cell number as measured by the protein content per well (Pierce 660 nM Protein Assay, Thermo Scientific).

### 2.6. Statistics 

Statistical evaluations were performed using Student’s paired *t*-tests, and differences were considered significant if *p* < 0.05. Data are presented as means +/– standard deviation (SD).

## 3. Results

### 3.1. Modulation of Wnt Pathway Gene Expression 

**Figure 1:**
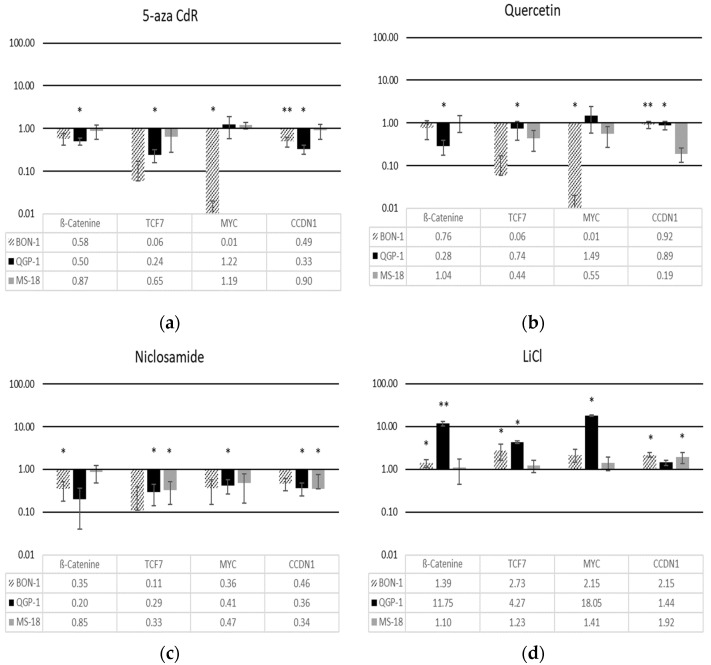
mRNA expression of ß-Catenin, TCF7, MYC, and CCDN1 (Cyclin D1) after treatment with (**a**) 5-aza CdR; (**b**) quercetin; (**c**) niclosamide; and (**d**) LiCl in BON-1, QGP-1, and MS-18 cells (logarithmic scale). The data are shown as percentages of untreated control cells. The expression levels were normalized to housekeeping gene GAPDH *(*mean ± SD; *n* = 6 experimental repeats; * *p* < 0.05, ** *p* < 0.01).

In the three cell lines, the effects of different Wnt-modulators on canonical Wnt signal transduction were characterized by qRT-PCR of the Wnt effector β-catenin and the Wnt target genes MYC, TCF7, and CCND1 (Figure 1).

We observed marked differences between the cell lines concerning the effects of Wnt modulators. In general, the three Wnt inhibitors 5-aza-CdR, quercetin, and niclosamide decreased β-catenin mRNA expression in BON-1 and QGP-1 cells, while the Wnt activator LiCl increased the expression of β-catenin. The latter effect was more pronounced in QGP-1 cells. In MS-18 cells, β-catenin mRNA expression was not affected by the Wnt modulators.

Depending on the cell line, the Wnt target genes were either downregulated or unchanged after treatment with the Wnt inhibitors. In BON-1 cells, all three inhibitors reduced Wnt target gene expression. This was most pronounced with 5-aza-CdR and quercetin, where TCF7 and MYC were markedly downregulated. In QGP-1 cells, 5-aza-CdR significantly downregulated TCF7 and CCND1 but did not change MYC expression. Quercetin did not affect the expression of these Wnt target genes in QGP-1 cells. In MS-18 cells, 5-aza-CdR did not affect TCF7, MYC, and CCND1 mRNA expression, while quercetin and niclosamide exposure led to a downregulation of those genes. Niclosamide was effective in reducing Wnt target genes in all three cell lines.

Treatment with the Wnt activator LiCl resulted in an upregulation of Wnt target genes (TCF7, MYC, and CCND1) in all three cell lines. The efficacy of LiCl was strongest in QGP-1 cells and weakest in MS-18 cells.

### 3.2. Effects of Wnt Pathway Modulation on CXCR4 Expression

The effects of the Wnt modulators on CXCR4 expression were analyzed by qRT-PCR, Western blot, and immunohistochemistry (IHC). When standardized for the housekeeper Glycerinaldehyd-3-phosphat-Dehydrogenase (GAPDH), basal CXCR4 mRNA expression was considerably higher in MS-18 cells as compared to the other cell lines (Supplementary Materials Figure S1).

**Figure 2:**
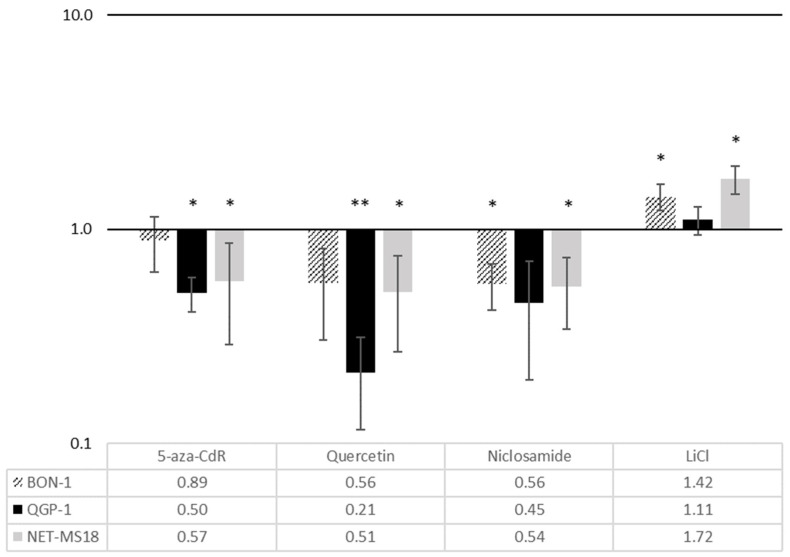
mRNA expression of CXCR4 after treatment with Wnt modulators in BON-1, QGP-1, and MS-18 cells (logarithmic scale). The data are shown as percentages of untreated control cells. The expression levels were normalized to housekeeping gene GAPDH *(*mean ± SD; *n* = 6 experimental repeats; * *p* < 0.05, ** *p* < 0.01).

All Wnt inhibitors reduced CXCR4 mRNA expression in BON-1, QGP-1, and MS-18 cells (Figure 2). This was most pronounced in QGP-1 cells, where the greatest effect was seen for quercetin (reduction by 79%, *p* < 0.01). The smallest effect on CXCR4 mRNA expression was observed in BON-1 cells after 5-aza-CdR exposure (reduction by 11%, *p* > 0.05). Stimulation with the Wnt activator LiCl significantly increased CXCR4 mRNA expression in BON-1 and MS-18 cells, with no influence on CXCR4 mRNA in QGP-1 cells.

**Figure 3:**
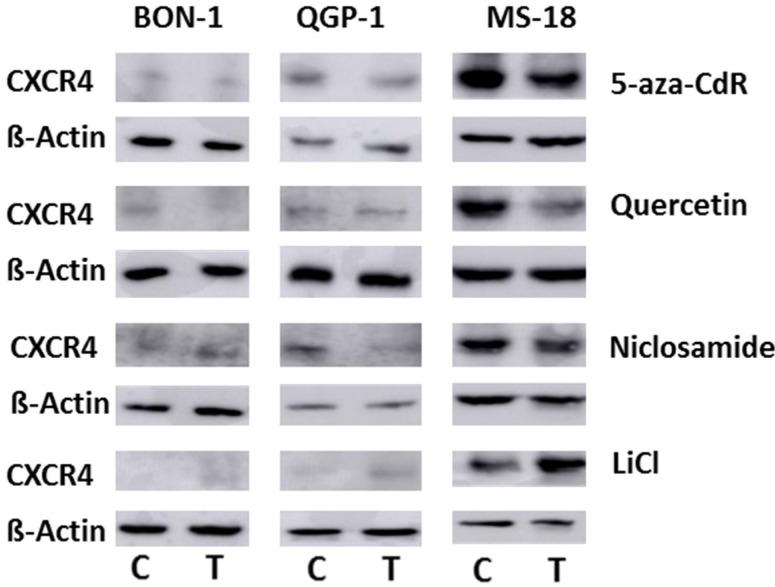
Immunoblot staining of CXCR4 in BON-1, QGP-1, and MS-18 either untreated (control) or after incubation with Wnt modulators. ß-Actin is exposed as the loading control.

Figure 3 shows the CXCR4 protein expression as determined by Western blot. In agreement with the RNA expression data, basal CXCR4 expression was considerably higher in MS-18 cells compared to the other cell lines, where faint bands suggested a weaker basal protein expression of CXCR4. Due to this higher CXCR4 expression in MS-18 cells, a fading of bands after Wnt inhibitor exposure and an increase in bands after exposure to the Wnt activator LiCl are visible. This is in line with the data from qRT-PCR.

**Figure 4:**
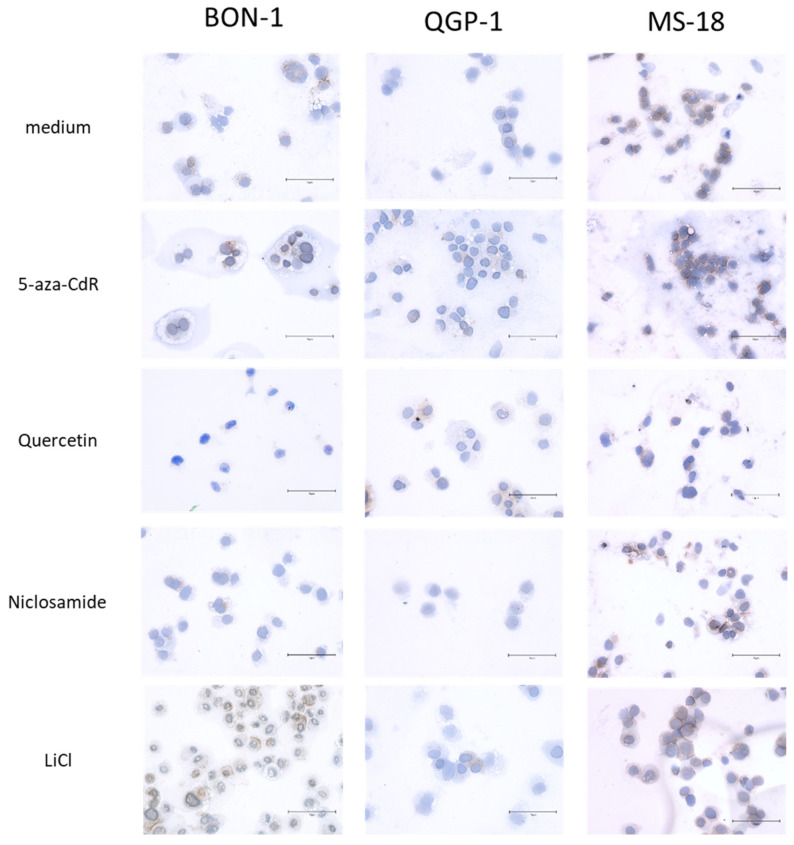
Immunohistochemistry staining of CXCR4 in BON-1, QGP-1, and MS-18 cells either before (control) or after incubation with Wnt modulators. The bar in the images represents 75 μm. The percentage of CXCR4-positive cells after immunohistochemical staining in BON-1, QGP-1, and MS-18 after Wnt modulator treatment is shown in Supplementary Materials Figure S2.

Immunohistochemical staining of CXCR4 was performed in all cell lines after incubation with the Wnt modulators (Figure 4). The strong basal expression of CXCR4 in MS-18 cells is again confirmed by the presence of cytoplasmic, membraneous, and nuclear staining, while basal staining was weak in BON-1 cells and QGP-1 cells. Upon exposure to Wnt inhibitors, CXCR4-specific staining decreased alongside the percentage of CXCR4-positive cells. As in Western blot, this effect was strongest with quercetin in MS-18 cells and least pronounced for 5-aza-CdR in MS-18 cells. Since there was very low basal CXCR4 staining in QGP-1 cells, the effect of Wnt inhibition in immunohistochemistry were only reflected by the percentage of CXCR4-positive cells (Supplementary Material Figure S2). After LiCl exposure, CXCR4 staining increased in BON-1 and MS-18 cells while remaining unaffected again in QGP-1 cells.

### 3.3. Effects of Wnt Modulators on [^68^Ga] Pentixafor Uptake

**Figure 5:**
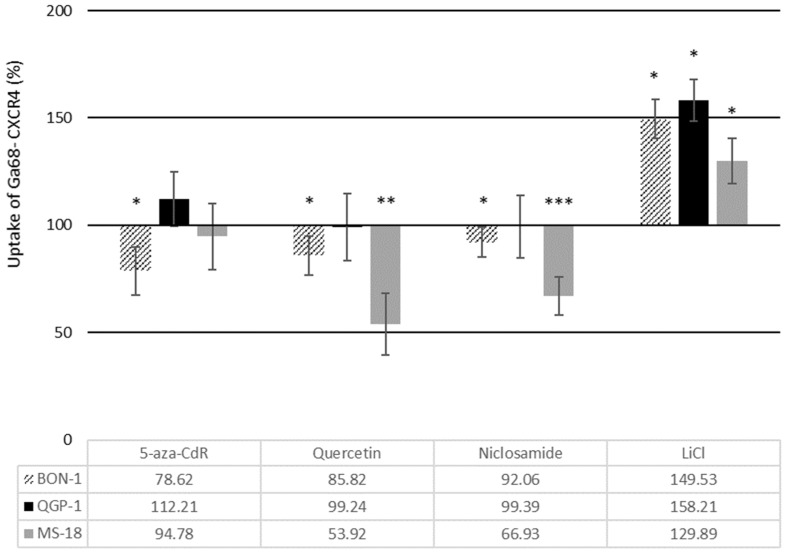
Specific uptake of radiolabeled [Ga68] Pentixafor in BON-1, QGP-1, and MS-18 cells after incubation with Wnt modulators. The data are presented as percentages of untreated controls (mean ± SD; *n* = 3 experimental repeats; * *p* < 0.05, ** *p* < 0.01, and *** *p* < 0.001).

To study the effects of Wnt modulation on CXCR4 function, the cells were incubated with the radioactively labeled CXCR4 preferring ligand [^68^Ga] Pentixafor with and without prior exposure to the Wnt modulators. Figure 5 shows the effects of the different drugs on specific uptake (internalization) of the radioligand compared to untreated controls. Incubation with Wnt inhibitors led to a significant decrease in [^68^Ga] Pentixafor uptake in BON-1 and MS-18 cells with the exception of 5-aza-CdR in MS-18 cells, where no significant effect was observed. This is in accordance with the IHC results but different from the observations made with qRT-PCR. In QGP-1 cells, [^68^Ga] Pentixafor uptake was mainly unaffected by the Wnt inhibitors. Exposure to the Wnt activator LiCl resulted in a marked increase in [^68^Ga] Pentixafor uptake in all three cell lines, which was most pronounced in QGP-1 cells (an increase of 58% from baseline, *p* < 0.05).

## 4. Discussion

Physiologically, the CXCL12-CXCR4 axis plays an important role in hematopoiesis, cardiogenesis, and neurogenesis [4,5,28]. Its pathological overexpression in various hematologic and solid neoplasms as well as in NEN was found to be associated with the acquisition of a mesenchymal phenotype favoring proliferation, invasion, and metastasis as well as a poorer prognosis when compared to tumors with low CXCR4 expression [4,5]. An inverse relationship between CXCR4 and SSTR2 expression was described in NEN, where increasing dedifferentiation is characterized by decreased SSTR2 expression and a rise in CXCR4 and Ki67 expression [3,13]. Treatment strategies directed against either CXCR4 expression or CXCR4-associated signal transduction may therefore be of interest for the treatment of dedifferentiated high-grade NEN when SSTR-directed therapies are not applicable. Understanding the mechanisms regulating CXCR4 expression and function would give further insight into the molecular processes defining the more aggressive phenotype of CXCR4-expressing neuroendocrine tumors and would help to enhance CXCR4-directed imaging and therapy.

Recently, Wnt/β-catenin activation in NET has been subject to increasing attention [29,30,31]. Abundant activation of the Wnt pathway also has been shown to favor cell migration, expression of EMT markers, and proliferation in neuroendocrine tumors in vitro [14,15]. The protooncogenes MYC, CCND1 (coding Cyclin D1, that is required for cell cycle progression), or TCF7 (coding TCF1, a nuclear mediator of β-catenin conveyed transcription) are target genes of the Wnt pathway and upregulated upon Wnt activation [32,33,34]. CXCR4 signaling employs Wnt signaling by inactivation of GSK3β via PI3K/Akt and stabilization of β-catenin. Stabilized β-catenin moves to the nucleus, activates gene transcription, and promotes proliferation [5]. On the other hand, a reciprocal mechanism where Wnt signaling regulates CXCR4 expression has been described in the postnatal dentate gyrus. In this model, it was shown that CXCR4 is a direct target gene of Lef1, a transcription factor of β-catenin-conveyed Wnt signal transduction [18].

Our data give evidence that pharmacological inhibition of Wnt signaling decreases CXCR4 expression and radioligand uptake in NET cells while Wnt stimulation increases CXCR4 expression and function.

For the NET cell models BON-1 and QGP-1, whole exome sequencing is available, describing no mutations in the CXCR4 gene or loss of heterozygosity on chromosome 2, where the CXCR4 gene is located [35,36]. Both cell lines share marked dedifferentiation but show considerable differences in the spectrum of mutations and chromosomal aberrations between them [35]. Mutations in Wnt pathway constituents have been described, which are differential between the two cell lines [35], suggesting a different degree of Wnt activation at baseline. As an example, a single-nucleotide variant in the APC gene was described in QGP-1 cells that was not present in BON-1 cells. In BON-1 cells, on the other hand, a single-nucleotide variant in the Wnt1 gene has been found [35]. This is also reflected by the differential baseline expression of β-catenin, TCF7, MYC, and CCND1 in BON-1, QGP-1, and MS-18 prior to treatment (Supplementary Figure S3). Cives et al. observed CXCR4 membrane expression in >25% of their BON-1 and QGP-1 cells by flow cytometry [16]. This is in line with our findings of low basal mRNA and protein expression of CXCR4 in these cell lines (Figure 3 and Figure 4, and Supplementary Materials Figures S1 and S3). Therefore, we included MS-18 cells, a primary cell culture recently derived from a rectal NEC that has a considerably higher basal expression of CXCR4 mRNA and protein in comparison to BON-1 and QGP-1 cells, into the experiment. MS-18 represents the CXCR4-expressing neuroendocrine carcinoma phenotype better than the other cell lines studied. In addition, the characteristics of the original tumor may be better preserved compared to the commonly used cell lines BON-1 and QGP-1, which have been in culture over a much longer period of time. A detailed characterization of MS-18 cells with whole-exome sequencing is not available yet but will be provided in the future.

Since each of the Wnt modulators used in our study may have other pharmacological properties apart from Wnt interference, we sought to include multiple drugs. All four substances studied are clinically used in humans and are known to interfere with the Wnt pathway at different levels of intracellular signal transduction [14,37,38,39].

We determined the EC50 for all drugs in both cell lines based on their inhibitory effect on cell growth. For all Wnt inhibitors, the concentrations used in our experiments were well below their EC50 and below plasma levels achieved in their therapeutic application (see the data in the Materials and Methods section). The mood stabilizer LiCl is a Wnt activator that stimulates proliferation in the cell cultures; thus, the in vitro EC50 was above the therapeutic plasma level for humans. Therefore, we chose a concentration that was above commonly used plasma levels and near the EC50 concentration in order to produce a definite effect on gene expression. Similarly, in a study on mesenchymal stem cells, LiCl affected CXCR4 only in doses above clinically used plasma levels [25,40].

In all cell lines, the Wnt inhibitors induced a downregulation of CXCR4 mRNA expression while treatment with the Wnt activator LiCl led to an upregulation. The strongest inhibitory effects on CXCR4 expression and function were found for quercetin.

Quercetin and niclosamide are Wnt inhibitors that also have been shown to reduce CXCR4 expression in breast cancer stem cells and in retinal microvascular pericytes [41,42]. The flavonoid quercetin is an attractive substance, since side effects are rare [43]. It has been shown to inhibit Wnt signaling by disrupting binding of the Tcf complex to its specific DNA-binding sites and to inhibit tumor growth of various malignancies in vitro [44]. It depressed CXCR4 mRNA expression in all three cell lines, having the strongest effect in QGP-1 with a downregulation by approximately 80% (*p* < 0.05). Niclosamide inhibits Wnt signaling by promoting Frizzled1 endocytosis, by downregulating Dishevelled-2 protein, and by inhibiting Wnt3A-stimulated beta-catenin stabilization as well as LEF/TCF reporter activity. In Bon-1, QGP-1, and MS-18 cells, niclosamide led to CXCR4 mRNA downregulation.

In our experiments, 5-aza-CdR showed the weakest effects on CXCR4 mRNA expression and radiopeptide uptake. This may be due to conflicting effects exerted by Wnt inhibition and CXCR4 promoter demethylation. The wide-ranging pharmacological effects of the synthetic nucleoside 5-aza-CdR include an increase in SSTR expression [20] and an inhibition of Wnt signaling [14] in NET cell lines. 5-aza-CdR was proposed to induce Wnt inhibition by restoring the function of Wnt inhibitory genes, e.g., SRFP-1 and WIF-1, by demethylation of their respective gene promoters or by histone demethylation, e.g., histone H3K9 [14]. Wnt inhibition would then downregulate CXCR4 expression. However, the effects of this demethylating agent on CXCR4 signaling may not be restricted to the Wnt pathway. In the breast cancer cell line MDA-MB-435, 5-aza-CDR reversed CXCR4 gene promoter hypermethylation, leading to a consecutive increase in CXCR4 expression [45].

LiCl has been shown to activate Wnt signaling and to upregulate CXCR4 mRNA expression by inhibiting by GSK-3 β [25,39]. In Bon-1, QGP-1, and MS-18, it induced an upregulation of CXCR4 mRNA expression and increase in radionuclide uptake.

So far, no information on [^68^Ga] Pentixafor uptake in BON-1, QGP-1, or MS-8 cells is available. As [^68^Ga] Pentixafor has a high affinity to CXCR4, the specific uptake reflects the amount of radiopeptide internalized by endocytosis after binding to functionally intact membrane-bound CXCR4 [10,11,12]. Treatment with the Wnt-activator LiCl led to an increase in specific [^68^Ga] Pentixafor uptake in all cell lines, suggesting a direct effect of Wnt activation or inhibition on CXCR4 function. In BON-1 and MS-18 cells, the Wnt inhibitors 5-aza-CdR, quercetin, and niclosamide decreased radiopeptide uptake, while in QGP-1 cells [^68^Ga] Pentixafor, uptake was not affected by Wnt inhibitor exposure. This is inconsistent with the qRT-PCR results, where we observed the strongest CXCR4 mRNA downregulation upon Wnt inhibitor exposure in QGP-1 cells. These differences between CXCR4 mRNA expression and the respective radiopeptide uptake might be due to the fact that receptor endocytosis and redirection are required for modulating CXCR4 signaling [46]. Therefore, an increased [^68^Ga] Pentixafor uptake does not necessarily need to be the result of an increased number of CXCR4 molecules on the cellular surface but may also be caused by an increase in turnover and redirection of the receptor to the membrane.

Since different Wnt inhibitors that interfere with the Wnt pathway at different levels exerted comparable direct effects on CXCR4 expression and since a Wnt activator had the opposite effect, the results strongly support a direct interaction of Wnt signaling with CXCR4 expression.

Because there is a general paucity of pancreatic NEN cell lines and most studies are limited to BON-1 and QGP-1 cells, we added the primary NEC cell line MS-18, which was recently established by our group and displays a higher basal CXCR4 expression. A limitation of the present study is that this cell line is not fully characterized at this time. Furthermore, this study only takes into account the canonical pathway while the effects of the used Wnt modulators might extend their effects on the noncanonical Wnt pathway or other intracellular signal transduction pathways.

## 5. Conclusions

Wnt pathway modulation affects CXCR4 expression and function in NET cell lines. In the dedifferentiated tumor cell lines used in our experiments, Wnt inhibitors inhibited CXCR4 expression and function while Wnt activation had the opposite effect. The exact underlying mechanisms need further investigation, but the notion that all cell lines responded indicates a more general applicability of Wnt modulators. Whether these results can be translated and reproduced in vivo in patients with dedifferentiated neuroendocrine tumors requires further investigation. The clinical goal would be to reduce CXCR4 receptor density by Wnt inhibition and therefore to decrease pro-proliferative and antiapoptotic signaling without unwanted hematopoietic stem cell mobilization. Moreover, short-time CXCR4 upregulation by simultaneous Wnt activation during ERT might result in higher radiopeptide uptake without increased radiation-related side effects. However, these perspectives must be validated in animal models and clinical studies.

## Data Availability

The data presented in this study are available in the article or supplementary material.

## Supplementary Materials

The following are available online at https://www.mdpi.com/2075-4418/11/2/367/s1, Supplementary Figure S1. Baseline CXCR4 (a) mRNA expression (**b**) Western blot (**c**) Ga^68^ Pentixafor uptake in BON-1, QGP-1 and MS-18 cells; bars show mean ± SEM from 6 experiments. Supplementary Figure S2. Percentage of CXCR4 positive cells after immunohistochemical staining in BON-1, QGP-1 and MS-18 after Wnt modulator treatment. Supplementary Figure S3. β-Catenin, TCF7, MYC, CCND1(CyclinD1) (a) mRNA expression (b) Western blot prior to treatment; bars show mean ± SEM from 6 experiments. Supplementary Table S1: List of Antibodies used for Western Blotting, Immunhistochemistry and TaqMan gene expression assays for qRT-PCR.
