# Supplementary material for: Wnt/β-Catenin Signaling Regulates CXCR4 Expression and [68Ga] Pentixafor Internalization in Neuroendocrine Tumor Cells
Source: Diagnostics (Basel). 2021 Feb 22;11(2):367. doi: 10.3390/diagnostics11020367 (PMC7926465; doi:10.3390/diagnostics11020367)
Supplement: Supplementary file 1 [file diagnostics-11-00367-s001.pdf]

Article

Wnt/β-catenin Signaling Regulates CXCR4 Expression and [^{68}Ga] Pentixafor Internalization in Neuroendocrine Tumor Cells

Alexander Weich [1,*], Dorothee Rogoll [1], Sophia Gawlas [1], Lars Mayer [2], Wolfgang Weich [1], Judit Pongracz [3], Theodor Kudlich [1], Alexander Meining [1] and Michael Scheurlen [1]

[1] Department of Internal Medicine II, Gastroenterology, University Hospital Würzburg, 97080 Würzburg, Germany; Rogoll_D@ukw.de (D.R.); gawlas_s@ukw.de (S.G.); wolfgang.weich@gmx.de (W.W.); kudlich_t@ukw.de (T.K.); meining_a@ukw.de (A.M.); Scheurlen_m@ukw.de (M.S.)

[2] Department of Nuclear Medicine, University Hospital Würzburg, 97080 Würzburg, Germany; mayer_l3@ukw.de

[3] Department of Pharmaceutical Biotechnology, Faculty of Pharmacy, University of Pecs, 7630 Pecs, Hungary; pongracz.e.judit@pte.hu

* Correspondence: weich_a@ukw.de; Tel.: +49–931–2014–4927

Citation: Weich, A.; Rogoll, D.; Gawlas, S.; Mayer, L.; Weich, W.; Pongracz, J.; Kudlich, T.; Meining, A.; Scheurlen, M. Wnt/β-catenin signaling regulates CXCR4 expression and [^{68}Ga] Pentixafor internalization in neuroendocrine tumor cells. *Diagnostics* **2021**, *11*, 367. https://doi.org/10.3390/diagnostics11020367

Academic Editor: Rudolf A. Werner

Received: 15 January 2021
Accepted: 16 February 2021
Published: 22 February 2021

Publisher's Note: MDPI stays neutral with regard to jurisdictional claims in published maps and institutional affiliations.

Copyright: © 2021 by the authors. Licensee MDPI, Basel, Switzerland. This article is an open access article distributed under the terms and conditions of the Creative Commons Attribution (CC BY) license (http://creativecommons.org/licenses/by/4.0/).

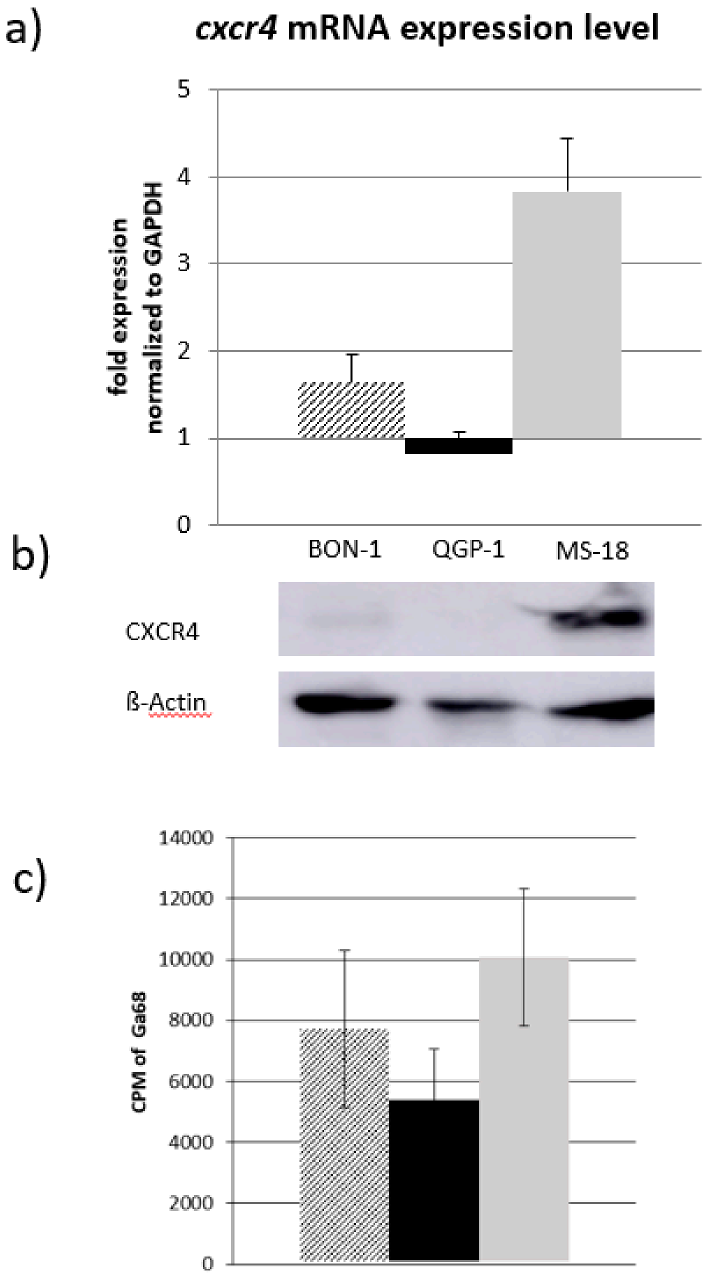

Supplementary Figure 1. Baseline CXCR4 **a)** mRNA expression **b)** Western blot **c)** Ga^{68} Pentixafor uptake in BON-1, QGP-1 and MS-18 cells; bars show mean ±SEM from 6 experiments.

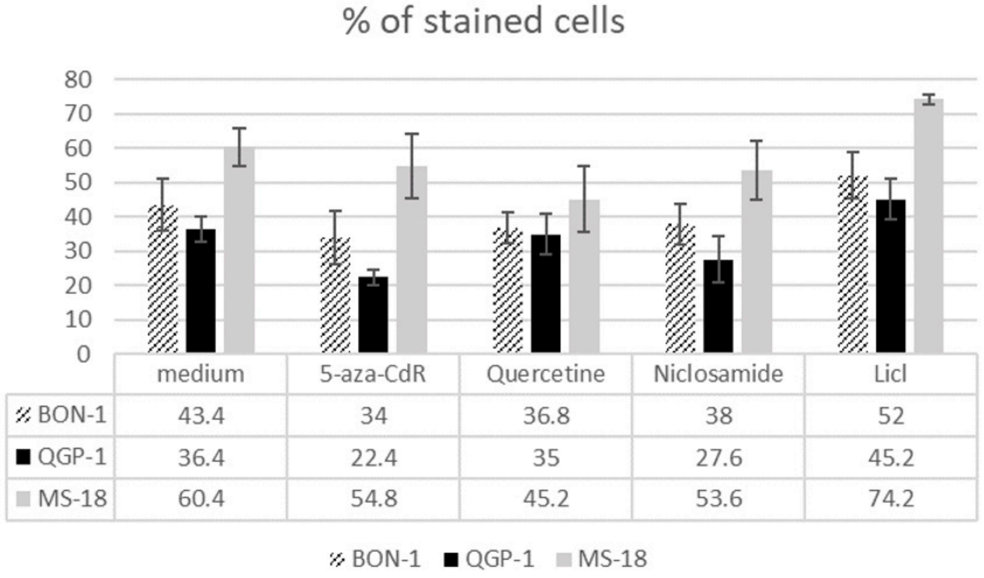

Supplementary Figure 2. Percentage of CXCR4 positive cells after immunohistochemical staining in BON-1, QGP-1 and MS-18 after Wnt modulator treatment.

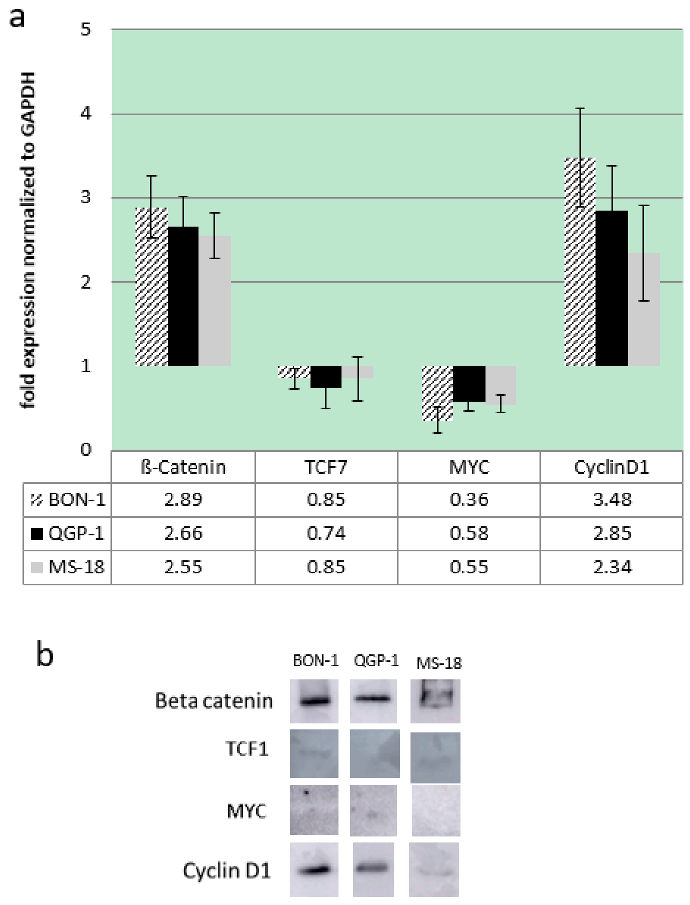

Supplementary Figure 3. β-Catenin, TCF7, MYC, CCND1(CyclinD1) **a)** mRNA expression **b)** Western blot prior to treatment; bars show mean ±SEM from 6 experiments.

gene symbol	*Western blot*	*second antibody*	*Immunhistochemistry*	*TaqMan assay ID applied biosystems*
CXCR4	ab124824	W401	ab124824	Hs00607978_s1
ß-Catenin	-	-	-	Hs00355049_m1
TCF7	-	-	-	Hs00175273_m1
MYC	-	-	-	Hs00153408_m1
CCND1	-	-	-	Hs00765553_m1
GAPDH	-	-	-	VIC/TAMRA #4310884E
ß-Actin	sc-47778	W401	-	-

Supplementary Table 1: List of Antibodies used for Western Blotting, Immunhistochemistry and TaqMan gene expression assays for qRT-PCR.
